# Recent Progress and Future Perspectives of MNb_2_O_6_ Nanomaterials for Photocatalytic Water Splitting

**DOI:** 10.3390/ma18153516

**Published:** 2025-07-27

**Authors:** Parnapalle Ravi, Jin-Seo Noh

**Affiliations:** Department of Semiconductor Physics, Gachon University, 1342 Seongnam-daero, Sujeong-gu, Seongnam-si 13120, Gyeonggi-do, Republic of Korea

**Keywords:** MNb_2_O_6_ photocatalyst, water splitting, visible-light activity, hydrogen evolution, heterostructure design

## Abstract

The transition to clean and renewable energy sources is critically dependent on efficient hydrogen production technologies. This review surveys recent advances in photocatalytic water splitting, focusing on MNb_2_O_6_ nanomaterials, which have emerged as promising photocatalysts due to their tunable band structures, chemical robustness, and tailored morphologies. The objectives of this work are to (i) encompass the current synthesis strategies for MNb_2_O_6_ compounds; (ii) assess their structural, electronic, and optical properties in relation to photocatalytic performance; and (iii) elucidate the mechanisms underpinning enhanced hydrogen evolution. Main data collection methods include a literature review of experimental studies reporting bandgap measurements, structural analyses, and hydrogen production metrics for various MNb_2_O_6_ compositions—especially those incorporating transition metals such as Mn, Cu, Ni, and Co. Novelty stems from systematically detailing the relationships between synthesis routes (hydrothermal, solvothermal, electrospinning, etc.), crystallographic features, conductivity type, and bandgap tuning in these materials, as well as by benchmarking their performance against more conventional photocatalyst systems. Key findings indicate that MnNb_2_O_6_, CuNb_2_O_6_, and certain engineered heterostructures (e.g., with g-C_3_N_4_ or TiO_2_) display significant visible-light-driven hydrogen evolution, achieving hydrogen production rates up to 146 mmol h^−1^ g^−1^ in composite systems. The review spotlights trends in heterojunction design, defect engineering, co-catalyst integration, and the extension of light absorption into the visible range, all contributing to improved charge separation and catalytic longevity. However, significant challenges remain in realizing the full potential of the broader MNb_2_O_6_ family, particularly regarding efficiency, scalability, and long-term stability. The insights synthesized here serve as a guide for future experimental investigations and materials design, advancing the deployment of MNb_2_O_6_-based photocatalysts for large-scale, sustainable hydrogen production.

## 1. Introduction

Ensuring a reliable and sustainable global energy supply constitutes one of the foremost scientific and technological challenges of the 21st century. As of 2008, global energy consumption reached approximately 15 terawatts (TW), and projections suggest that this demand could nearly double by 2050 due to continued population growth and industrial expansion [1]. A range of alternative energy sources—including solar, wind, hydropower, and geothermal—offer more environmentally benign and sustainable options compared to conventional fossil fuels. Nevertheless, the integration of these renewable resources into the energy infrastructure presents significant challenges owing to their intrinsic limitations. Geothermal energy, for instance, is constrained by high operational costs and finite site lifespans. In detail, geothermal energy is a clean, reliable baseload power source, but its wider adoption is limited by high capital and operational costs. Installation costs range from USD 2000 to 6000 per kW, with drilling alone making up over half of this. A single well can cost up to USD 10 million, and exploratory failures increase expenses. Annual operation and maintenance add USD 150–190 per kW, with reinjection accounting for 12–15% of costs in some plants. The levelized cost of electricity (LCOE) rose from about USD 0.049/kWh in 2010 to USD 0.071/kWh in 2020, exceeding USD 0.10/kWh in difficult locations, making it less competitive than solar or wind. Geothermal plants typically maintain efficiency for 25–30 years, with reservoir declines of 0.5–1% annually. For instance, Kenya’s Aluto–Langano plant’s output fell from 7.3 MW to under 1 MW over 20 years due to reservoir degradation [2,3,4,5,6]. These issues highlight the need for innovation and cost-cutting measures to ensure geothermal energy’s long-term viability. Hydropower is associated with considerable environmental impacts and the substantial capital investment required for dam construction. Wind energy, while abundant and clean, suffers from intermittency and the lack of cost-effective large-scale energy storage solutions [7].

Hydrogen (H_2_) is considered an environmentally benign energy carrier and has garnered significant attention in response to the depletion of conventional fossil fuels such as petroleum and coal [8,9]. Among the various methods for hydrogen production, photocatalytic water splitting stands out as an efficient and sustainable approach, as it harnesses solar energy—a freely available and abundant resource globally. Consequently, hydrogen holds considerable promise as a clean energy source for the future, given its non-toxic nature and its potential to be produced from readily available natural resources like water and sunlight. These sources are both renewable and environmentally sustainable [10]. A wide range of semiconductor materials has been investigated for photocatalytic H_2_ evolution [11], with titanium dioxide (TiO_2_) remaining one of the most extensively studied candidates due to its advantageous attributes—namely, non-toxicity, robust photochemical stability, and economic viability [10]. Despite these merits, TiO_2_ is intrinsically limited by its wide bandgap (~3.2 eV), which restricts photoresponse to the ultraviolet (UV) region, comprising less than 5% of the solar spectrum. Moreover, the material exhibits a high electron–hole recombination rate, which further constrains its photocatalytic efficiency. To overcome these deficiencies, considerable effort has been directed toward heterojunction engineering, wherein TiO_2_ is coupled with narrow bandgap semiconductors capable of visible-light absorption and improved charge carrier separation. This strategy enhances solar light utilization and suppresses recombination losses, thereby improving overall photocatalytic performance [12,13,14]. Among the emerging visible-light-active semiconductors, graphitic carbon nitride (g-C_3_N_4_) has attracted significant attention. This metal-free polymeric material features a moderate bandgap of approximately 2.7 eV, making it suitable for harnessing visible light. In addition to its favorable optoelectronic properties, g-C_3_N_4_ exhibits notable thermal and chemical stability and can be synthesized via straightforward thermal polycondensation processes using low-cost precursors such as urea or melamine [15,16,17]. Thermogravimetric analysis studies consistently demonstrate that g-C_3_N_4_ is thermally stable up to approximately 600 °C in air or nitrogen atmospheres, with significant decomposition occurring only above this temperature [18,19]. For example, g-C_3_N_4_ materials synthesized from dicyandiamide or melamine exhibit pronounced stability until around 600 °C, after which gradual thermal degradation takes place. Further studies confirm that the material maintains its structural integrity under ambient and moderate high-pressure conditions up to 600–700 °C, with decomposition to graphite and nitrogen observed only above these thresholds. This high decomposition temperature underscores the notable thermal stability of g-C_3_N_4_ and supports its advantageous application as a robust photocatalyst in various high-temperature processes. These characteristics render g-C_3_N_4_ a compelling component in TiO_2_-based heterojunction systems, offering synergistic effects that advance the design of next-generation photocatalysts for sustainable hydrogen production. This pivotal discovery has spurred the development of a wide range of novel inorganic semiconductor photocatalysts aimed at enhancing H_2_ production efficiency. These materials include strontium titanate (SrTiO_3_) [20], zinc sulfide (ZnS) [21], niobates [22,23], TiO_2_ [24,25,26], CdS [12], cuprous oxide (Cu_2_O) [27], zinc oxide (ZnO) [28], bismuth ferrite (BiFeO_3_) [29], lanthanum ferrite (LaFeO_3_) [30], perovskite-type metal oxides [13,31], as well as tin dioxide (SnO_2_) [32], tungsten trioxide (WO_3_) [33], hematite (Fe_2_O_3_) [34], niobium pentoxide (Nb_2_O_5_) [23], vanadium pentoxide (V_2_O_5_) [35], and tantalum pentoxide (Ta_2_O_5_) [26,36]. The majority of these materials have band gaps greater than 3.0 eV, meaning they can only respond to ultraviolet (UV) light. Given that UV light constitutes only a small portion of the solar spectrum, visible-light-active materials are required to produce a greater amount of hydrogen.

In recent years, several review articles have been published focusing on hydrogen production through photocatalytic water splitting. For instance, the review by Acar et al. provides a comprehensive overview of various photocatalytic water-splitting methods and discusses key parameters such as bandgap engineering, performance evaluation, and chemical recycling strategies [37]. Similarly, Corredor et al. examined the influence of different system components on photocatalytic performance, specifically analyzing the roles of sacrificial agents, catalysts, photosensitizers, and solvents in hydrogen evolution [38]. Another notable review addresses a broader range of renewable energy sources, with a dedicated section on photocatalytic hydrogen production [39]. It highlights aspects such as the structural and optical properties of photocatalysts, photostability, the role of sacrificial reagents, recent technological developments, and limitations associated with photodetection systems.

However, this review offers a detailed discussion on narrow band gap materials of the AB_2_O_6_ family. In particular, transition metal niobates with the general formula MNb_2_O_6_ (M = Cu, Ni, Mg, Zn, Co, Fe, Mn., etc.) represent a class of emerging photocatalysts due to their favorable electronic structure, chemical stability, and visible-light activity. These materials typically exhibit orthorhombic or monoclinic crystal structures, with tunable bandgaps ranging from ~2.0 to 3 eV depending on the transition metal cation. Their tunable electronic structures, relative chemical stability, and visible light absorption capabilities make them promising candidates to replace conventional wide bandgap materials.

## 2. Methodology

This review was conducted through an extensive survey of the peer-reviewed literature on MNb_2_O_6_ nanomaterials, focusing on their synthesis, structural and optical properties, and photocatalytic water-splitting performance. Relevant studies were collected from databases such as ScienceDirect, Web of Science, Scopus, SpringerLink, and Google Scholar using targeted keywords including “MNb_2_O_6_,” “photocatalysis,” “water splitting,” and “hydrogen evolution.” Selection criteria included experimental or theoretical studies published between 2000 and 2025 that reported on the synthesis, band structure, photocatalytic activity, and compositional modifications of MNb_2_O_6_ materials. Key parameters, such as synthesis methods, bandgap energies, photocatalytic H_2_ evolution rates, and material structures, were extracted, compared, and critically analyzed. The goal was to provide a clear summary of the current state of research, identify promising compounds, highlight underexplored materials, and propose future directions for improving MNb_2_O_6_-based photocatalysts for solar-driven hydrogen generation.

## 3. Synthetic Approaches for MNb_2_O_6_ Nanomaterials

The synthesis of MNb_2_O_6_ compounds has been achieved through a variety of methods, each offering specific advantages in terms of morphology control, crystallinity, and scalability. Key methods employed in the literature are summarized below.

### 3.1. Chemical Transport Method

Early structural investigations of MNb_2_O_6_ compounds, including NiNb_2_O_6_, were performed using chemical transport methods. This approach involves transporting precursor materials in the vapor phase using agents such as Cl_2_ within a two-zone or vertical furnace system. The method enabled the growth of high-quality single crystals, suitable for detailed crystallographic and optical studies [40].

### 3.2. Solid-State Reaction

The solid-state reaction remains a widely used conventional method for synthesizing polycrystalline MNb_2_O_6_ materials. Typically, metal oxides (e.g., NiO, CuO) and Nb_2_O_5_ are mixed in stoichiometric ratios and subjected to high-temperature calcination. This method is often used as a precursor step for single crystal growth techniques like the floating zone method, which provides large, defect-minimized crystals for fundamental studies [41].

### 3.3. Solvothermal and Hydrothermal Methods

Low-temperature solution-based techniques, such as solvothermal and hydrothermal synthesis, have gained attention for producing MNb_2_O_6_ nanostructures with controlled morphology and phase purity. In solvothermal routes, metal chlorides and NbCl_5_ are dissolved in organic solvents and reacted in sealed autoclaves at elevated temperatures. Hydrothermal methods, by contrast, utilize aqueous basic media under similar thermal conditions to generate nanocrystals or microstructured powders. Reaction parameters such as temperature, duration, pH, and precursor ratios play crucial roles in determining the final product’s particle size and surface area—factors critical for photocatalytic efficiency [42,43].

### 3.4. Electrospinning

Electrospinning, followed by thermal treatment, has been employed to fabricate one-dimensional nanofibers of MNb_2_O_6_ materials such as CoNb_2_O_6_. This technique combines a polymer-based spinning solution with applied voltage to generate continuous fibers, which are then annealed to obtain crystalline oxide nanofibers. This method offers the advantage of producing high-aspect-ratio nanostructures with interconnected porous frameworks, beneficial for charge transport in photocatalysis [44].

## 4. Structural Features of MNb_2_O_6_ Nanomaterials

**Figure 1:**
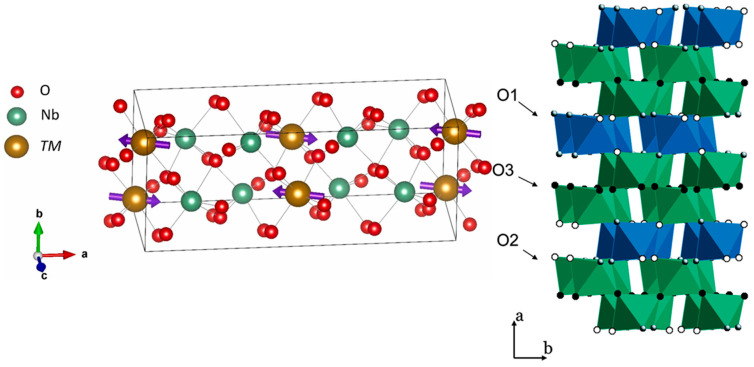
The crystal structure of columbite-type MNb_2_O_6_, highlighting the distinct oxygen sites: O1 (gray spheres), O2 (black spheres), and O3 (white spheres). Copyright 2023, Elsevier.

MNb_2_O_6_ compounds commonly crystallize in distorted columbite-type structures (orthorhombic, space group *Pbcn*) or related monoclinic phases. The columbite-type structure remains stable for various divalent transition metal ions such as Co, Fe, and Cu in the MNb_2_O_6_ framework [42,45,46], allowing for the tuning of electronic conductivity by selecting specific M-site cations or their combinations. As illustrated in Figure 1, the columbite crystal structure [47,48] comprises layers of slightly distorted hexagonal close-packed oxygen octahedra oriented perpendicular to the a-axis. Within each b–c plane, cation-occupied octahedra form zigzag chains running along the c-axis, connected through shared edges. Along the a-axis, the octahedrally coordinated cations follow a periodic sequence of M–Nb–Nb–M–Nb–Nb–M, contributing to the anisotropic nature of the crystal structure and influencing both structural and electronic properties. As shown in Figure 1, the columbite structure includes three distinct types of oxygen coordination environments: O1, which is coordinated to two Nb atoms and one M atom (represented as gray spheres); O2, which is bonded to one Nb and two M atoms (white spheres); and O3, which is shared among three Nb-centered octahedra (black spheres). These variations in oxygen coordination play a crucial role in defining the local bonding environment and influence electronic and catalytic behavior.

The M-site cations occupy interstitial positions between the NbO_6_ frameworks and influence the overall distortion and connectivity of the octahedra. In orthorhombic columbite structures, the Nb atoms form chains of edge-sharing octahedra along one crystallographic axis. The degree of distortion in the NbO_6_ units varies depending on the size and electronic configuration of the M cation. This structural flexibility allows for tunable electronic and optical properties, making MNb_2_O_6_ materials suitable for photocatalytic applications. Additionally, the presence of M^2+^/M^3+^ cations can introduce localized electronic states, which affect band structure and charge separation dynamics. The distinguished crystallographic parameters for MNb_2_O_6_ (M = Fe, Cu, Mg, Zn, Co, Mn, and Ni) ceramics are summarized in Table 1. All samples are crystallized in a single-phase columbite structure with the same *Pbcn* space group [49].

## 5. Morphological Features of MNb_2_O_6_ Nanomaterials

The morphology of MNb_2_O_6_ nanomaterials plays a pivotal role in determining their photocatalytic efficiency, particularly for water-splitting applications. Key morphological attributes such as particle size, shape, aspect ratio, crystallinity, and surface area directly affect light absorption, charge carrier mobility, and surface reaction kinetics.

Various synthesis strategies have been employed to tailor these morphological features (Table 2). For instance, Lee et al. synthesized MnNb_2_O_6_, CuNb_2_O_6_, and ZnNb_2_O_6_ nanoparticles via a solvothermal method, producing uniformly distributed nanocrystals with high surface areas, which facilitated visible-light-driven photocatalytic activity [42]. Similarly, Chun et al. prepared ZnNb_2_O_6_ nanocrystals using a hydrothermal route, achieving rod-like morphologies that enhanced light scattering and improved surface reactivity [43]. One-dimensional (1D) morphologies have shown particular promise due to their enhanced charge transport properties. Park et al. synthesized CoNb_2_O_6_ nanofibers through electrospinning followed by thermal annealing, resulting in a fibrous network with improved conductivity and extended photogenerated charge carrier lifetimes [44].

Moreover, hollow or porous structures and hierarchical assemblies of MNb_2_O_6_ are being actively explored, as they provide high surface-to-volume ratios, short diffusion paths, and more exposed reactive facets. Such morphological engineering is vital for maximizing the active interface between the photocatalyst and the electrolyte, thereby enhancing water-splitting efficiency. Despite these advances, most studies have focused on only a few MNb_2_O_6_ compounds, such as MnNb_2_O_6_ and CoNb_2_O_6_. Systematic morphological studies on other members of this family (e.g., MgNb_2_O_6_, FeNb_2_O_6_, NiNb_2_O_6_) remain limited. Additionally, there is a need for correlative studies linking specific morphological traits to photocatalytic performance metrics to better inform rational design strategies.

In summary, tailoring the morphology of MNb_2_O_6_ nanomaterials is essential for optimizing their photocatalytic behavior. Future research should emphasize morphology–performance relationships across the broader MNb_2_O_6_ family, using advanced characterization techniques such as SEM, TEM, BET, and AFM, alongside performance testing under standard photocatalytic conditions.

## 6. Optical Properties of MNb_2_O_6_ Compounds

A critical factor influencing the photocatalytic performance of MNb_2_O_6_ materials is their band gap energy, which determines their light absorption capability. We have compiled and compared available experimental band gap values with theoretical calculations to provide a comprehensive overview.

Experimental band gaps of MNb_2_O_6_ compounds typically fall within the visible to near-UV range. For instance, MnNb_2_O_6_ exhibits band gaps between 2.3 and 2.7 eV, making it active under visible light [42]. CuNb_2_O_6_ shows somewhat narrower band gaps around 1.9–2.1 eV, which correlates with its strong photocatalytic and photoelectrochemical activities [51]. CoNb_2_O_6_′s band gap is around 2.4 eV, supporting its visible-light-driven hydrogen evolution capabilities [44]. NiNb_2_O_6_ presents band gaps from 2.2 to 2.9 eV, consistent with its reported photocatalytic hydrogen production under UV-visible irradiation [52].

In contrast, ZnNb_2_O_6_, MgNb_2_O_6_, and FeNb_2_O_6_ exhibit wider band gaps ranging from approximately 3.6 to 4.0 eV, restricting their absorption primarily to the UV region [43,50]. These values align with their observed photocatalytic activities, mainly for UV-assisted processes rather than visible-light water splitting.

Theoretical band gap calculations obtained via density functional theory (DFT) tend to slightly overestimate experimental values but remain in good agreement overall. For example, DFT predicts band gaps of ~2.5–2.8 eV for MnNb_2_O_6_ and ~2.0–2.2 eV for CuNb_2_O_6_, closely matching experimental measurements. Similarly, theoretical values for ZnNb_2_O_6_ and MgNb_2_O_6_ are about 3.7–4.2 eV, consistent with experimental data (Table 3).

This comparison not only validates theoretical models but also reinforces the suitability of particular MNb_2_O_6_ compounds (e.g., MnNb_2_O_6_, CuNb_2_O_6_, CoNb_2_O_6_, NiNb_2_O_6_) for visible-light-driven photocatalytic water-splitting applications. The wider band gap materials may require further modifications such as doping or heterojunction formation to enhance visible light absorption and overall photocatalytic efficiency.

**Figure 2:**
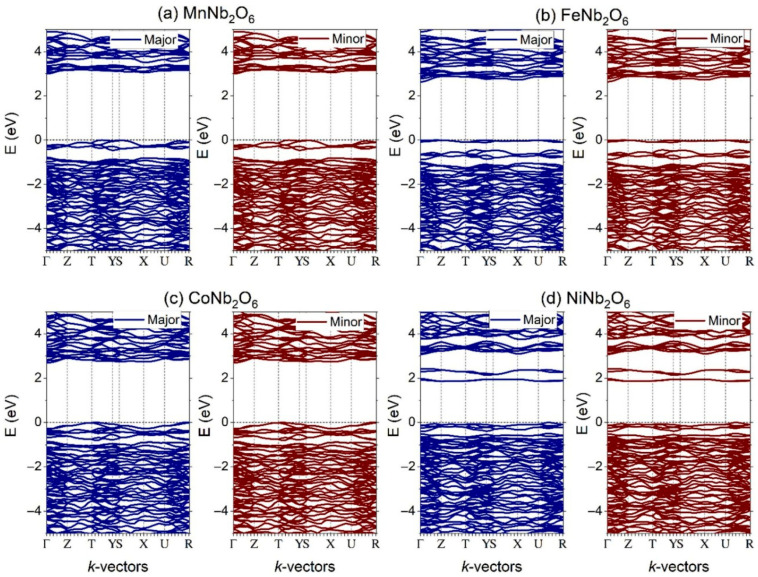
The electronic band structures of (**a**) MnNb_2_O_6_ (**b**) FeNb_2_O_6_, (**c**) CoNb_2_O_6_, and (**d**) NiNb_2_O_6_ nanomaterials. Copyright 2023, Elsevier.

The band structures of all the compounds studied by Naveed-Ul-Haq et.al, obtained through GGA + U calculations and plotted along high-symmetry paths (Figure 2a–d), reveal wide insulating band gaps ranging from 2.64 to 2.98 eV. In comparison, experimental band gap values have been reported as 2.21–2.48 eV for MnNb_2_O_6_ [56] and 2.42 eV for CoNb_2_O_6_ [57]. NiNb_2_O_6_ shows a notably lower band gap of 1.86 eV, which closely aligns with the 1.98 eV reported by Karmakar et al. [58]. Notably, the band gap remains consistent across both the majority and minority spin channels. The Fermi level is positioned at the top of the valence band, suggesting a p-type conductivity character for these materials.

**Figure 3:**
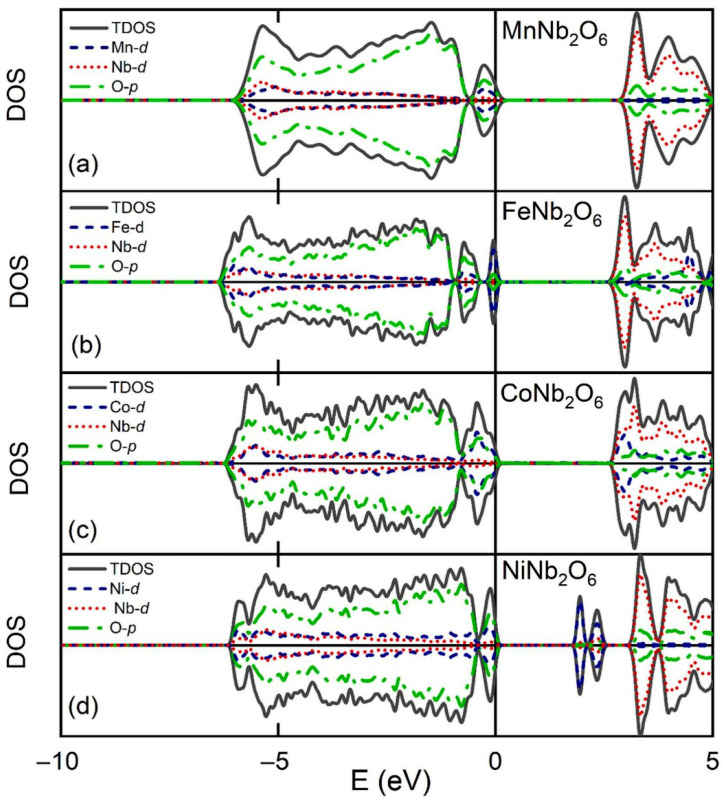
Total and element-resolved density of states (DOS) plots for (**a**) MnNb_2_O_6_ (**b**) FeNb_2_O_6_, (**c**) CoNb_2_O_6_, and (**d**) NiNb_2_O_6_. Copyright 2023, Elsevier.

Figure 3a–d present the total density of states (TDOS) along with the element-resolved partial density of states (PDOS) for the investigated compounds. In MnNb_2_O_6_, CoNb_2_O_6_, and NiNb_2_O_6_, the valence band (VB) edge is predominantly composed of O 2p orbitals, with smaller contributions from the d-orbitals of the respective transition metals (Mn, Co, and Ni). In contrast, for FeNb_2_O_6_, the upper VB edge is mainly dominated by Fe 3d states, with only minimal involvement from O 2p orbitals. On the conduction band (CB) side, the lower edge in both MnNb_2_O_6_ and FeNb_2_O_6_ is primarily shaped by Nb 4d states, accompanied by minor contributions from O 2p states. For CoNb_2_O_6_, the Co 3d orbitals contribute more significantly to the CB minimum compared to the Mn and Fe counterparts. Uniquely, in NiNb_2_O_6_, the lower VB edge is almost entirely formed by Ni 3d states, differing from the other compounds. All the materials exhibit an antiferromagnetic ground state, with no net magnetic moment, as the spins of the transition metal ions (Mn, Fe, Co, and Ni) are aligned in an antiparallel configuration [54,59].

Moreover, the oxygen vacancies are prevalent point defects in niobates and typically act as donor states, shifting the Fermi level closer to the conduction band and thereby favoring n-type conductivity. In contrast, copper-based niobates may exhibit p-type behavior due to the involvement of Cu 3d orbitals in hole generation; their defect chemistry and behavior in related systems suggest that Mg, Fe, Ni, Zn, and Mn variants are predominantly n-type (Table 4). Co and Cu substitutions, however, may introduce more complex or ambipolar conduction characteristics.

## 7. Photocatalytic Performance of MNb_2_O_6_ Compounds

The MNb_2_O_6_ family, where M represents divalent or transition metals such as Mn, Fe, Co, Ni, Mg, Cu, and Zn, exhibits a rich variety of structural and optical properties, positioning these materials as attractive candidates for photocatalytic water splitting. However, only a subset of these materials has demonstrated significant activity under experimental conditions, highlighting both the potential and the gaps in our current understanding.

### 7.1. Visible-Light-Active Niobates: MnNb_2_O_6_, CuNb_2_O_6_, and NiNb_2_O_6_

Among the MNb_2_O_6_ compounds, MnNb_2_O_6_, CuNb_2_O_6_, and NiNb_2_O_6_ are particularly promising due to their moderate band gaps (~2.2–2.9 eV), which enable visible-light absorption.

MnNb_2_O_6_-based Composites: Several studies have explored MnNb_2_O_6_ integrated with g-C_3_N_4_ or porous morphologies to enhance photocatalytic activity. For instance, a MnNb_2_O_6_/g-C_3_N_4_ binary system synthesized via solvothermal and sonochemical methods achieved degradation efficiencies over 94% for various antibiotics under visible light, indicating strong potential for water purification and solar energy applications. High-surface-area MnNb_2_O_6_ also demonstrated enhanced performance in photodegradation tasks.

**Figure 4:**
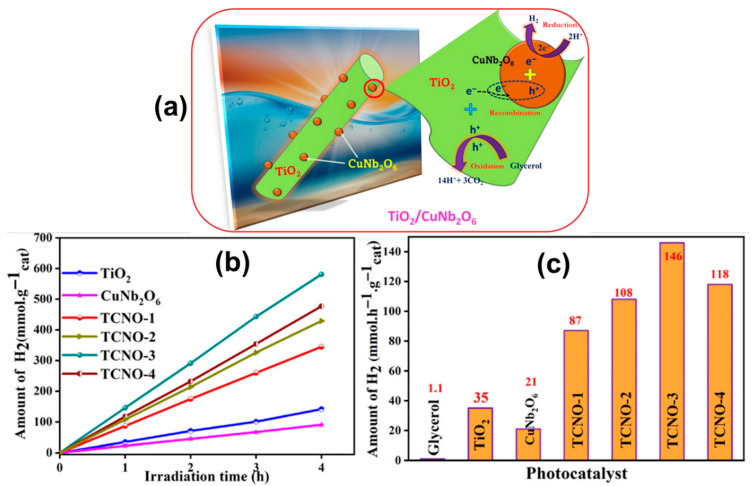
(**a**) Schematic mechanism of TiO_2_/CuNb_2_O_6_ system, (**b**) H_2_ evolution with time, and (**c**) average rate of H_2_ production. Reprinted with permission from ref. [50]. Copyright ©2023 American Chemical Society.

CuNb_2_O_6_ Photocatalysis: CuNb_2_O_6_ has been used both as a standalone material and in composites. Li et al. fabricated p-type CuNb_2_O_6_ via spray pyrolysis for use as a photocathode in photoelectrochemical cells. CuNb_2_O_6_/g-C_3_N_4_ and CuNb_2_O_6_ polymorph studies revealed that monoclinic phases exhibit superior photocatalytic behavior. Further, CuNb_2_O_6_-based heterojunctions, such as TiO_2_/CuNb_2_O_6_, significantly enhanced hydrogen evolution rates, with performance reaching 146 mmol h^−1^ g^−1^ in optimized composites (Figure 4). In detail, the structural analysis by X-ray diffraction (XRD) confirms the successful formation of the TiO_2_/CuNb_2_O_6_ composite, exhibiting distinct peaks corresponding to both anatase TiO_2_ and orthorhombic CuNb_2_O_6_ phases with no impurity signals, which indicates good crystallinity and effective interfacial contact. Morphological studies using SEM and TEM reveal that CuNb_2_O_6_ nanoparticles are uniformly anchored on the TiO_2_ surface, providing a high density of well-connected interfacial sites that facilitate efficient charge transfer and limit electron–hole recombination. Optical analysis, based on diffuse reflectance UV-Vis spectroscopy, shows that the composite’s band gap is significantly narrowed (2.26–2.77 eV) compared to pure TiO_2_ (~3.2 eV), resulting in extended visible light absorption up to around 410 nm. This improved visible-light harvesting capability is directly linked to the composite’s enhanced photocatalytic activity [60].

**Figure 5:**
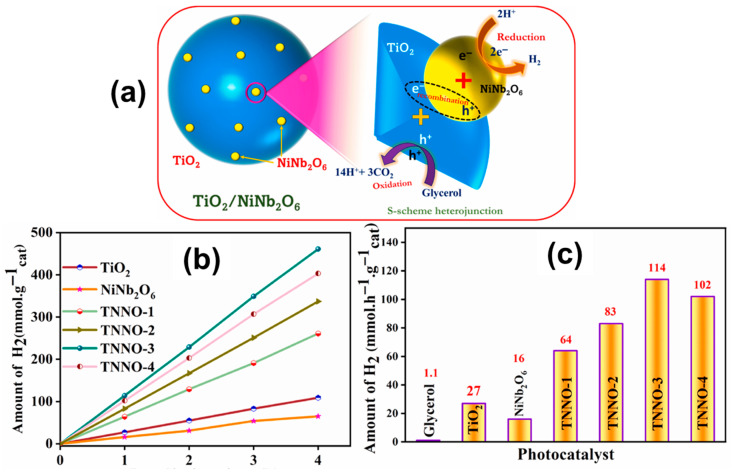
(**a**) Schematic mechanism of TiO_2_/NiNb_2_O_6_ system, (**b**) H_2_ evolution with time, and (**c**) average rate of H_2_ production. Copyright 2023, Elsevier.

NiNb_2_O_6_ Heterojunctions and Solid-State Systems: NiNb_2_O_6_ has been investigated in heterostructures with reduced graphene oxide (RGO) and exfoliated g-C_3_N_4_ (E-gC_3_N_4_), showing efficient degradation of pollutants and enhanced photocatalytic activity under visible and sunlight irradiation. S-scheme heterojunctions like TiO_2_/NiNb_2_O_6_ composites improved charge separation, achieving hydrogen production rates up to 114 mmol h^−1^ g^−1^. Solid-state synthesized NiNb_2_O_6_ also showed promising hydrogen evolution under visible light without co-catalysts, with a band gap around 2.9 eV (Figure 5). In detail, XRD analysis confirms the coexistence of both TiO_2_ and NiNb_2_O_6_ phases in the composite, indicating successful synthesis. SEM and TEM images show that NiNb_2_O_6_ is well-dispersed on the TiO_2_ surface, resulting in good interfacial contact. Optical measurements reveal a reduced band gap for the composite compared to pure TiO_2_, enabling enhanced absorption of visible light and improved photocatalytic activity [52].

### 7.2. CoNb_2_O_6_: Emerging Potential Through Heterostructure Engineering

CoNb_2_O_6_ has shown measurable hydrogen evolution activity when integrated into heterostructures, such as with TiO_2_ in S-scheme composites. These engineered systems benefit from improved charge separation and extended light absorption, leading to enhanced photocatalytic performance. Despite this progress, direct evidence of H_2_ production from pure CoNb_2_O_6_ without composite engineering remains scarce. Satiya et al. reported the design and fabrication of a novel 2D/2D CoNb_2_O_6_/g-C_3_N_5_ type-II heterojunction photocatalyst aimed at enhancing visible-light-driven degradation of cefuroxime, a commonly used antibiotic and emerging water pollutant. The composite structure was engineered to maximize interfacial contact between the layered CoNb_2_O_6_ and g-C_3_N_5_, promoting efficient charge separation and transfer across the heterojunction. This synergistic interaction significantly improved the photocatalytic performance under visible light irradiation [61]. In a recent study, Mohammad et al. investigated the electrochemical properties of trirutile-phase CoNb_2_O_6_ microspheres and reported their pseudocapacitive behavior governed by anion intercalation mechanisms. This form of charge storage, which involves reversible insertion/extraction of anions within the electrode structure, significantly enhances the material’s capacitive performance. Furthermore, the authors demonstrated that CoNb_2_O_6_ exhibits considerable electrocatalytic activity toward water-splitting reactions, indicating its dual functionality as both an energy storage medium and a catalyst for sustainable energy applications [62].

### 7.3. Limited Exploration: ZnNb_2_O_6_, MgNb_2_O_6_, and FeNb_2_O_6_

Experimental data on MgNb_2_O_6_ and FeNb_2_O_6_ remain scarce for photocatalytic water splitting, although their structural properties have been characterized.

**Figure 6:**
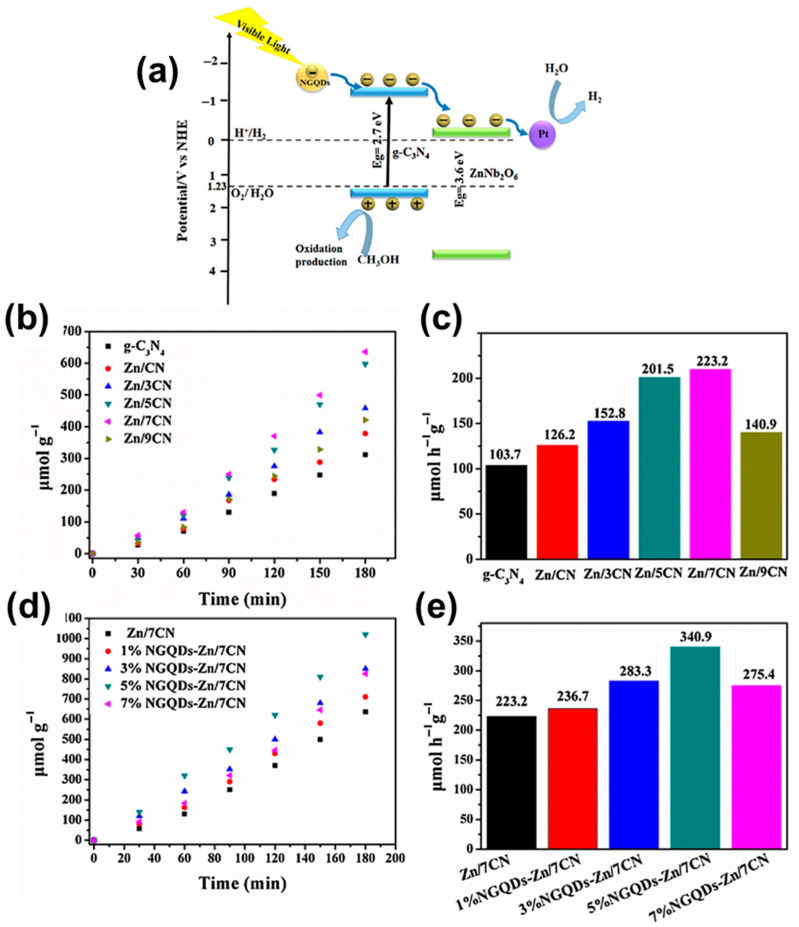
(**a**) Possible mechanism occurring in g-C_3_N_4_/ZnNb_2_O_6_. Photocatalytic hydrogen evolution profiles of various samples under visible light irradiation (λ > 420 nm) as a function of time (**b**,**d**). Comparative analysis of hydrogen evolution rates under visible light illumination for the different samples (**c**,**e**). Copyright 2017, Elsevier.

**Figure 7:**
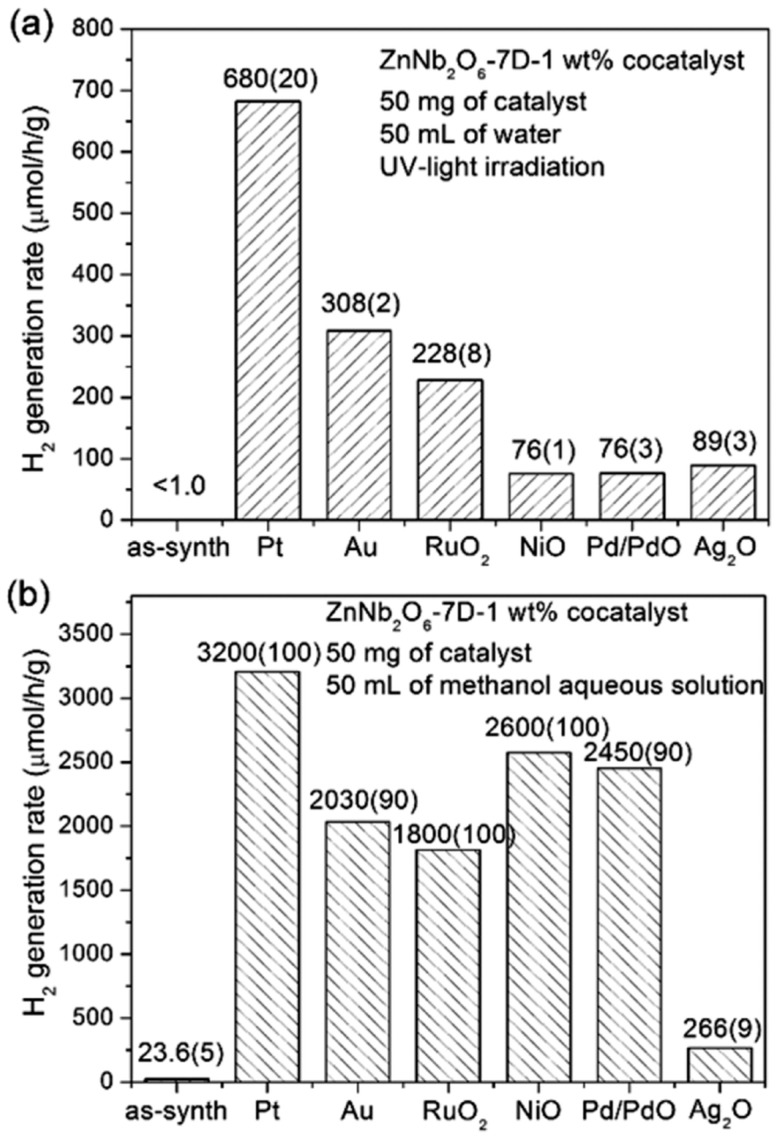
Photocatalytic hydrogen evolution rates of ZnNb_2_O_6_ synthesized over 7 days (ZnNb_2_O_6_–7D) loaded with 1 wt% cocatalyst, measured under UV light irradiation in (**a**) pure water and (**b**) a 20 vol% methanol aqueous solution. (Open access).

ZnNb_2_O_6_ Systems: ZnNb_2_O_6_ has a wide band gap (~3.8 eV), restricting activity to UV light. However, composites like ZnNb_2_O_6_/g-C_3_N_4_ integrated with nitrogen-doped graphene quantum dots (NGQDs) have improved visible-light hydrogen evolution. Optimized heterojunctions showed rates of 340.9 µmol h^−1^ g^−1^ (Figure 6). Furthermore, noble metal-loaded ZnNb_2_O_6_ (e.g., with Pt or Ag) significantly enhanced hydrogen generation, achieving up to 3200 µmol h^−1^ g^−1^ in methanol–water systems (Figure 7).

MgNb_2_O_6_ Applications: While MgNb_2_O_6_ is not yet established for water splitting, it has shown promise in dye degradation under UV light. Recent work on Dy- and Er-doped MgNb_2_O_6_ materials has highlighted their wide bandgap (~4–5 eV) and moderate dye-removal efficiencies, confirming their suitability for pollutant degradation.

Finally, MnNb_2_O_6_, NiNb_2_O_6_, CoNb_2_O_6_, and CuNb_2_O_6_ have shown the most promising hydrogen evolution rates under visible light, attributed to their optimal band gaps (2.1–2.9 eV), well-ordered crystal structures, and favorable morphologies. On the other hand, MgNb_2_O_6_, FeNb_2_O_6_ and ZnNb_2_O_6_ lack detailed photocatalytic hydrogen production reports. Their structural and morphological features suggest potential, but the absence of systematic hydrogen evolution studies represents a clear research gap.

## 8. Influence of Specific Surface Area on the Photocatalytic Performance of MNb_2_O_6_ Nanomaterials

The specific surface area of MNb_2_O_6_ nanomaterials, as determined by BET (Brunauer–Emmett–Teller) analysis, plays a crucial role in influencing their photocatalytic water-splitting performance. A higher specific surface area provides a greater number of active sites for surface redox reactions, enhances the adsorption of water and sacrificial agents, and facilitates efficient charge separation by shortening the migration paths of photogenerated carriers. For example, in the TiO_2_/CuNb_2_O_6_ composite reported by Ravi et al., the BET surface area increased from ~91 m^2^/g for pure CuNb_2_O_6_ to ~132 m^2^/g for the composite. This enhancement corresponded with a significant improvement in hydrogen evolution rate from ~21 mmol g^−1^ h^−1^ to ~146 mmol g^−1^ h^−1^ under visible light. Similarly, the g-C_3_N_4_/ZnNb_2_O_6_ heterojunction developed by Yan et al. exhibited a surface area of ~56.8 m^2^/g compared to ~18.9 m^2^/g for pure ZnNb_2_O_6_, resulting in an increased H_2_ generation rate of ~340 µmol g^−1^ h^−1^. In both cases, the elevated surface area enabled better light absorption, enhanced reactant diffusion, and reduced electron–hole recombination. These results underscore that tailoring the surface area through heterostructure formation and nanostructuring is a viable strategy to improve the photocatalytic efficiency of MNb_2_O_6_ materials, making BET analysis a vital tool in photocatalyst optimization. The comparative Table 5 below summarizes the structural, morphological features, synthesis methods, surface areas, and photocatalytic hydrogen production performance of various MNb_2_O_6_ compounds.

Among the MNb_2_O_6_ family, Mn, Cu, Ni, and Co-based compounds have shown the most promise for visible-light photocatalytic hydrogen evolution. Their performance is largely dependent on material morphology, composite design, and the engineering of heterojunctions that facilitate charge separation. Conversely, Mg, Fe, and Zn-based niobates require further exploration to unlock their full potential. Future work should prioritize systematic comparisons, cocatalyst optimization, and exploration of doped and hybrid systems across the full MNb_2_O_6_ family to advance sustainable hydrogen generation technologies.

## 9. Research Gaps in Advancing MNb_2_O_6_-Based Photocatalysts

MnNb_2_O_6_ and CuNb_2_O_6_ have emerged as promising candidates for photocatalytic water splitting due to their suitable bandgap energies and responsiveness to visible light. CoNb_2_O_6_ has also demonstrated measurable hydrogen evolution under solar irradiation, particularly when incorporated into heterostructured composites that enhance charge separation and light-harvesting efficiency. However, in contrast to these encouraging findings, other members of the MNb_2_O_6_ family—such as MgNb_2_O_6_, FeNb_2_O_6_, NiNb_2_O_6_, and ZnNb_2_O_6_—remain largely unexplored in terms of their direct photocatalytic hydrogen production. This gap underscores the need for systematic experimental studies to assess and unlock their full potential.

Although several MNb_2_O_6_ compounds have shown promise for photocatalytic water splitting, significant knowledge gaps persist across this material family. Notably, NiNb_2_O_6_, MnNb_2_O_6_, and CuNb_2_O_6_ have demonstrated favorable photocatalytic hydrogen evolution performance, attributed to their appropriate band gap energies (~2.0–2.9 eV) and visible-light responsiveness. CoNb_2_O_6_ has also been reported to exhibit appreciable hydrogen production under solar irradiation, particularly when incorporated into heterostructured composites that enhance charge separation and light harvesting.

In contrast, limited experimental data are available for other MNb_2_O_6_ members such as MgNb_2_O_6_, FeNb_2_O_6_, and ZnNb_2_O_6_. These materials have primarily been explored for UV-assisted dye degradation or dielectric applications, with few or no reports evaluating their direct hydrogen evolution performance. Even in cases where structural and optical properties have been characterized, systematic assessment of their photocatalytic behavior—especially under visible light—remains absent.

Moreover, there is a lack of studies exploring band structure engineering, heterojunction formation, or surface modification strategies for the lesser-known MNb_2_O_6_ phases. Without such investigations, the full photocatalytic potential of these materials remains untapped. In addition, few reports provide detailed insight into charge carrier dynamics or employ advanced electronic characterization techniques such as Mott–Schottky analysis, Hall-effect measurements, or Seebeck coefficient evaluation to validate theoretical predictions and guide material optimization.

Furthermore, comparative studies across the MNb_2_O_6_ series are notably scarce. Most available reports investigate single compounds under varying experimental conditions, limiting the ability to derive meaningful structure–property relationships or performance trends.

In summary, although select MNb_2_O_6_ compounds exhibit promising photocatalytic activity, comprehensive and systematic studies across the broader material family are critically needed. Addressing these gaps through coordinated experimental efforts, advanced characterization, and rational design strategies will be essential for unlocking the full potential of MNb_2_O_6_-based nanomaterials in sustainable hydrogen production.

## 10. Conclusions

This review highlights the recent progress in MNb_2_O_6_-based nanomaterials for photocatalytic water splitting, emphasizing their structural, morphological, optical, and performance characteristics. Among the reported compounds, narrow-bandgap MNb_2_O_6_ compounds such as MnNb_2_O_6_, CuNb_2_O_6_, NiNb_2_O_6_, and CoNb_2_O_6_ offer significant visible-light absorption (band gaps, 2.0–2.9 eV), directly translating to improved solar-to-hydrogen efficiency. Notably, optimized TiO_2_/CuNb_2_O_6_ and TiO_2_/NiNb_2_O_6_ composites achieved H_2_ evolution rates of up to 146 mmol h^−1^ g^−1^ and 114 mmol h^−1^ g^−1^, respectively, while g-C_3_N_4_/ZnNb_2_O_6_ heterojunctions reached 340 μmol h^−1^ g^−1^ under visible light—outperforming many conventional oxide photocatalysts. Structurally, MNb_2_O_6_ compounds typically adopt a columbite-type framework, which offers flexibility for electronic and morphological modifications. Morphological analysis reveals a strong correlation between surface area and activity; for instance, the hydrogen evolution rate for TiO_2_/CuNb_2_O_6_ increased substantially—from 21 to 146 mmol h^−1^ g^−1^—as the BET surface area was enhanced from ~91 to 132 m^2^/g. Additionally, strong alignment between theoretical and experimental band gap values supports the rational design of MNb_2_O_6_-based photocatalysts for visible light applications. Despite notable progress in Mn-, Cu-, Ni-, and Co-based systems, limited studies are available on MgNb_2_O_6_, FeNb_2_O_6_, and ZnNb_2_O_6_ for overall water splitting. These materials are often restricted to UV-driven dye degradation, and their potential under visible light remains underexplored. Unlike broader reviews, this review presents the first focused comparison of the MNb_2_O_6_ family, compiling quantitative data on structural, optical, and photocatalytic performance metrics. It also emphasizes the role of heterojunction construction, defect engineering, and surface area optimization in enhancing photocatalytic outcomes. Looking ahead, research should focus on (i) the development of underexplored MNb_2_O_6_ phases and their heterostructures, (ii) detailed investigations linking defect structures, charge carrier dynamics, and morphology to activity, and (iii) the adoption of scalable synthesis methods suited for practical applications. Standardized benchmarking of photocatalytic metrics will also be essential for identifying structure–property–performance relationships across the MNb_2_O_6_ series. In conclusion, MNb_2_O_6_ compounds represent a versatile and promising class of visible-light-active photocatalysts. Bridging current knowledge gaps through integrated experimental and computational studies will be key to advancing their application in sustainable hydrogen production.

## Figures and Tables

**Table 1:** Structural parameters of MNb_2_O_6_ transition metal niobates.

Compound	Space Group	a (Å)	b (Å)	c (Å)	Notes on Structure
MgNb_2_O_6_ (columbite)	Pbcn	14.1875	5.7001	5.0331	NbO_6_ octahedra form edge-sharing 1D chains along the c-axis; MgO_6_ connects via corners (ui.adsabs.harvard.edu)
FeNb_2_O_6_ (columbite)	Pbcn	~14.281	5.7366	5.1234	Details from single-crystal studies; demonstrates full orthorhombic symmetry
NiNb_2_O_6_ (columbite)	Pbcn	14.032	5.687	5.033	Prepared as single crystals; consistent with columbite-type structure
ZnNb_2_O_6_ (columbite)	Pbcn	14.208	5.726	5.040	In line with earlier findings, edge-sharing NbO_6_ chains are evident
MnNb_2_O_6_	Pbcn	14.39	5.74	5.09	Powder, orthorhombic columbite; a = 0.5766 nm, b = 1.4439 nm, c = 0.5085 nm (V = 0.4234 nm^3^) (akjournals.com)
CoNb_2_O_6_	Pbcn	~14.18	~5.72	~5.04	Orthorhombic columbite phase confirmed
CuNb_2_O_6_	Pbcn	14.10	5.70	5.03	Analogous orthorhombic parameters expected; comparable to other MNb_2_O_6_ columbites

**Table 2:** Morphological features of MNb_2_O_6_ nanomaterials and their photocatalytic implications.

Compound	Morphology	Synthesis Method	Key Features & Observations	Reference
MnNb_2_O_6_	Nanoparticles	Solvothermal	Uniform dispersion, high surface area; enhances visible-light absorption and H_2_ evolution	[42]
CuNb_2_O_6_	Nanoparticles	Solvothermal	Small particle size; suitable bandgap for photocatalysis	[42]
ZnNb_2_O_6_	Nanorods	Hydrothermal	Rod-like morphology improves charge transport and light harvesting	[43]
CoNb_2_O_6_	Nanofibers	Electrospinning + Annealing	1D structure enhances electron mobility; hierarchical fibrous network improves separation	[44]
FeNb_2_O_6_	Micro-/nanoparticles	Solid-state/Sol–gel	Limited reports on photocatalytic water splitting; morphology less controlled	[46]
NiNb_2_O_6_	Crystals, bulk powder	Solid-state/Floating Zone	Mostly studied for magnetic/dielectric properties; photocatalytic morphology underexplored	[40]
MgNb_2_O_6_	Irregular particles	Solid-state	Rarely explored for photocatalysis; lack of controlled morphology studies	[50]

**Table 3:** Comparison of experimental and theoretical band gap values for various MNb_2_O_6_ compounds, along with their synthesis methods and corresponding literature references. The synthesis approach influences the morphology and crystallinity of the materials, thereby affecting their optical and photocatalytic properties.

Compound	Experimental Band Gap (eV)	Theoretical Band Gap (eV)	Synthesis Method	Key References
MnNb_2_O_6_	2.3–2.7	2.5–2.8	Solid-state reaction	[53]
CuNb_2_O_6_	1.9–2.1	2.0–2.2	Sol–gel method	[51]
CoNb_2_O_6_	~2.4	2.3–2.5	Hydrothermal method + annealing	[44]
NiNb_2_O_6_	2.2–2.5	2.4–2.7	Solid-state reaction	[54]
ZnNb_2_O_6_	3.6–3.8	3.7–3.9	Hydrothermal method	[55]
MgNb_2_O_6_	~3.9	4.0–4.2	Sol–gel auto-combustion	[50]
FeNb_2_O_6_	~4.0	4.0–4.2	Solid-state reaction	[54]

**Table 4:** Bandgap nature of MNb_2_O_6_ transition metal niobates.

Compound	Conductivity Type	Reasoning/Evidence
MgNb_2_O_6_	Likely n-type	Analogous dielectric niobates tend to be n-type due to oxygen vacancies (pmc.ncbi.nlm.nih.gov, reddit.com); Mg has no open d-orbitals.
FeNb_2_O_6_	Likely n-type	Transition-metal niobates often show n-type behavior from oxygen-vacancy donors.
NiNb_2_O_6_	Likely n-type	Similar logic: oxygen deficiencies act as electron donors in Nb–O systems.
ZnNb_2_O_6_	Predominantly n-type	Widely used as a dielectric; typical oxide defects (e.g., O-vacancies) lead to n-type conduction.
MnNb_2_O_6_	Likely n-type, possibly p-type	No direct measurement. Structurally similar oxides are n-type, but Mn d-states could allow minority hole conduction.
CoNb_2_O_6_	Likely n-type, possible mixed behavior	No direct data. Cobalt oxides can sometimes be p-type, but niobate structure suggests n-type dominance.
CuNb_2_O_6_	Potentially p-type, possibly ambipolar	Cu^2+^ often introduces hole carriers; certain copper niobates show p-type or bipolar conductivity (needs experimental confirmation).

**Table 5:** Structural phases, morphologies, synthesis routes, surface areas, and hydrogen production performances of selected MNb_2_O_6_ nanomaterials. Note that data are based on available experimental reports and indicate that while MnNb_2_O_6_ and CuNb_2_O_6_ demonstrate promising photocatalytic activity, other members of the MNb_2_O_6_ family remain underexplored in the context of hydrogen evolution.

Compound	Crystal Structure (Space Group)	Morphology	Synthesis Method	Surface Area (m^2^/g)	H_2_ Evolution Rate (μmol h^−1^ g^−1^)	Reference
MnNb_2_O_6_	Orthorhombic (*Pbcn*)	Rod-like nanoparticles	Hydrothermal	~28.3	Not reported	[53]
CuNb_2_O_6_	Orthorhombic (*Pbcn*)	Plate-like microstructures	Sol-gel	~91	~21,000	[60,63]
CoNb_2_O_6_	Orthorhombic	Nanofibers in composites	Electrospinning + Calcination	~12.7	~190 (in heterojunction)	[62]
MgNb_2_O_6_	Orthorhombic	Irregular granules	Solid-state reaction	Not reported	Not reported	[50]
FeNb_2_O_6_	Monoclinic	Agglomerated particles	Solid-state reaction	~5.1	Not reported	[54]
NiNb_2_O_6_	Monoclinic	Dense particles	Solid-state reaction	~96	16,000	[52]
ZnNb_2_O_6_	Orthorhombic	Porous, nanoscale aggregates	Hydrothermal	~18.9	23	[43]

## Data Availability

The data used to support the findings of this study are available from the corresponding author upon request.
